# Supplementary material for: A randomized trial looking at planning prompts to reduce opioid prescribing
Source: Nat Commun. 2024 Jan 12;15:263. doi: 10.1038/s41467-023-44573-5 (PMC10786898; doi:10.1038/s41467-023-44573-5)
Supplement: Supplementary file 1 — Supplementary Information [file 41467_2023_44573_MOESM1_ESM.pdf]

Supplementary Methods

S1. Standard Letter

Date

Dear *Prescriber Name, Degree*,
This letter is to inform you that your patient, *Decedent Name*, *DOB,* died on *DOD.* Opioid overdose was either the primary cause of death or contributed to the death.

The Los Angeles County Medical Examiner's Office typically sees approximately 500 opioid-related deaths each year. A significant proportion of deaths are due to the combination of multiple prescription medications. Patients may obtain legitimate prescriptions for opioids, benzodiazepines, muscle relaxants, and sleep aids (non-benzodiazepine sedative/hypnotics) from more than one prescriber. When taken in any combination, these medications put patients at greater risk of overdose and death. We also see many deaths that are a result of long-term therapeutic prescribing.

Our aim is to alert providers to the potential dangers of opioid medications and how common death from misuse of these medications is in Los Angeles County.

The Los Angeles County Medical Examiner and the County Health Officer would like to remind you that California has a prescription drug monitoring program called the Controlled Substance Utilization Review and Evaluation System (CURES), which *helps prescribers who are dedicated to avoiding prescribing controlled substances when those substances, despite being clinically appropriate, are likely to do more harm than good.*

The CURES database contains information about whether and when other providers have prescribed controlled substances to your patient within the last 12 months only. **Review of this database before prescribing a scheduled medication to a patient could alert you to problematic medication usage or potential addiction or diversion, and help promote safe medication prescribing and monitoring.** As of October 2, 2018, prescribers are required to consult CURES prior to prescribing, ordering, administering, or furnishing a Schedule II-IV controlled substances (CA HSC § 11165.4).

This database and access to information are available at https://cures.doj.ca.gov/. If needed, you can register for CURES at https://cures.doj.ca.gov/registration/confirmEmailPnDRegistration.xhtml.

The following evidence-based interventions also lower overdose death rates:
1. **Avoid concurrent prescribing of an opioid and a benzodiazepine, non-benzodiazepine sedative/hypnotic, or muscle relaxant.** Opioids and these other medications can have additive central nervous system depressant effects.
2. **Avoid opioid prescribing as a first-line, routine, or lone therapy for chronic non-cancer pain and avoid/minimize opioid prescribing for acute pain.** According to the

Centers for Disease Control and Prevention (CDC), providers should avoid opioids as a first-line, routine, or lone therapy for chronic pain outside of active cancer treatment, palliative care, and end-of-life care. When used for chronic pain, follow-up and re-evaluate pain, functioning, and risk of harm. When necessary for acute pain, start with the lowest effective dose of immediate-release opioids. Three days or less is often sufficient to address acute pain. Opioids should not be considered first-line or routine therapy for chronic pain.

3. **Safely taper opioids to safer doses**. For patients already on long-term high-dose opioid therapy, the CDC recommends tapering to a dose that is lower than 50 morphine milligram equivalents (MME) and that slow opioid tapers as well as pauses in the taper may be needed for long-term users.

4. **Offer Medications for Addiction Treatment (MAT), as appropriate.** Ensure individuals with an alcohol and/or opioid use disorder are offered MAT, when clinically appropriate, either directly or through a facilitated referral to an addiction specialist.

5. **Avoid "the 90-day cliff."** One California study[1] found that nearly 70% of patients who died of prescription-related overdoses were prescribed the same medication for 3 consecutive months. The CDC recommends that clinicians should consider how opioids will be discontinued if benefits do not outweigh risks to patient safety if realistic goals for pain and function have not been met.

6. **The CDC recommends prescribing naloxone to patients on 50 MME/day or higher of opioids.**

Again, this letter is meant to be informative only, is not affiliated with other initiatives, and there is no expectation of reply. We understand that learning of your patient's death can be difficult. We hope that you will take this as an opportunity to join us in preventing future deaths from drug overdose.

Jonathan Lucas, M.D.
Chief Medical Examiner-Coroner

Muntu Davis, M.D., M.P.H.
County Health Officer

---

[1] Lev, R., Petro, S., Lee, O., Lucas, J., Stuck, A., Vilke, G. M., & Castillo, E. M. (2016). A description of Medical Examiner prescription-related deaths and prescription drug monitoring program data. Am J Emerg Med, 34(3), 510-514.

S2. Comparator Letter

Date

Dear *Prescriber Name, Degree*,

This letter is to inform you that your patient, *Decedent Name*, *DOB,* died on *DOD.* Opioid overdose was either the primary cause of death or contributed to the death.

The Los Angeles County Medical Examiner's Office typically sees approximately 500 opioid-related deaths each year. A significant proportion of deaths are due to the combination of multiple prescription medications. Patients may obtain legitimate prescriptions for opioids, benzodiazepines, muscle relaxants, and sleep aids (non-benzodiazepine sedative/hypnotics) from more than one prescriber. When taken in any combination, these medications put patients at greater risk of overdose and death. We also see many deaths that are a result of long-term therapeutic prescribing.

Our aim is to alert providers to the potential dangers of opioid medications and how common death from misuse of these medications is in Los Angeles County.

The Los Angeles County Medical Examiner and the County Health Officer would like to remind you that California has a prescription drug monitoring program called the Controlled Substance Utilization Review and Evaluation System (CURES), which *helps prescribers who are dedicated to avoiding prescribing controlled substances when those substances, despite being clinically appropriate, are likely to do more harm than good.*

The CURES database contains information about whether and when other providers have prescribed controlled substances to your patient within the last 12 months only. **Review of this database before prescribing a scheduled medication to a patient could alert you to problematic medication usage or potential addiction or diversion, and help promote safe medication prescribing and monitoring.** As of October 2, 2018, prescribers are required to consult CURES prior to prescribing, ordering, administering, or furnishing a Schedule II-IV controlled substances (CA HSC § 11165.4).

This database and access to information are available at https://cures.doj.ca.gov/. If needed, you can register for CURES at https://cures.doj.ca.gov/registration/confirmEmailPnDRegistration.xhtml.

The following evidence-based interventions also lower overdose death rates:

1. **Avoid concurrent prescribing** of an opioid and a benzodiazepine, non-benzodiazepine sedative/hypnotic, or muscle relaxant. Opioids and these other medications can have additive central nervous system depressant effects.
2. **Avoid opioid prescribing as a first-line, routine, or lone therapy for chronic non-cancer pain and avoid/minimize opioid prescribing for acute pain.** According to the Centers for Disease Control and Prevention (CDC), providers should avoid opioids

as a first-line, routine, or lone therapy for chronic pain outside of active cancer treatment, palliative care, and end-of-life care. When used for chronic pain, follow-up and re-evaluate pain, functioning, and risk of harm. When necessary for acute pain, start with the lowest effective dose of immediate-release opioids. Three days or less is often sufficient to address acute pain. Opioids should not be considered first-line or routine therapy for chronic pain.

3. **Safely taper opioids to safer doses.** For patients already on long-term high-dose opioid therapy, the CDC recommends tapering to a dose that is lower than 50 morphine milligram equivalents (MME) and that slow opioid tapers as well as pauses in the taper may be needed for long-term users.
4. **Offer Medications for Addiction Treatment (MAT), as appropriate.** Ensure individuals with an alcohol and/or opioid use disorder are offered MAT, when clinically appropriate, either directly or through a facilitated referral to an addiction specialist.
5. **Avoid "the 90-day cliff."** One California study[1] found that nearly 70% of patients who died of prescription-related overdoses were prescribed the same medication for 3 consecutive months. The CDC recommends that clinicians should consider how opioids will be discontinued if benefits do not outweigh risks to patient safety if realistic goals for pain and function have not been met.
6. **The CDC recommends prescribing naloxone** to patients on 50 MME/day or higher of opioids.

When your next patient presents with pain, keep the above 6 recommendations close at hand to assist with their safe care. Also, be comfortable voicing your concern about prescribing safety with them so that they are also aware of the dangers associated with scheduled drugs. We also encourage you to routinely log into CURES and learn more about the prescriptions your patient received leading up to the death.

Again, this letter is meant to be informative only, is not affiliated with other initiatives, and there is no expectation of reply. We understand that learning of your patient's death can be difficult. We hope that you will take this as an opportunity to join us in preventing future deaths from drug overdose.

Jonathan Lucas, M.D.
Chief Medical Examiner-Coroner

Muntu Davis, M.D., M.P.H.
County Health Officer

---

[1] Lev, R., Petro, S., Lee, O., Lucas, J., Stuck, A., Vilke, G. M., & Castillo, E. M. (2016). A description of Medical Examiner prescription-related deaths and prescription drug monitoring program data. Am J Emerg Med, 34(3), 510-514.

Supplementary Tables and Figures

Fig S1

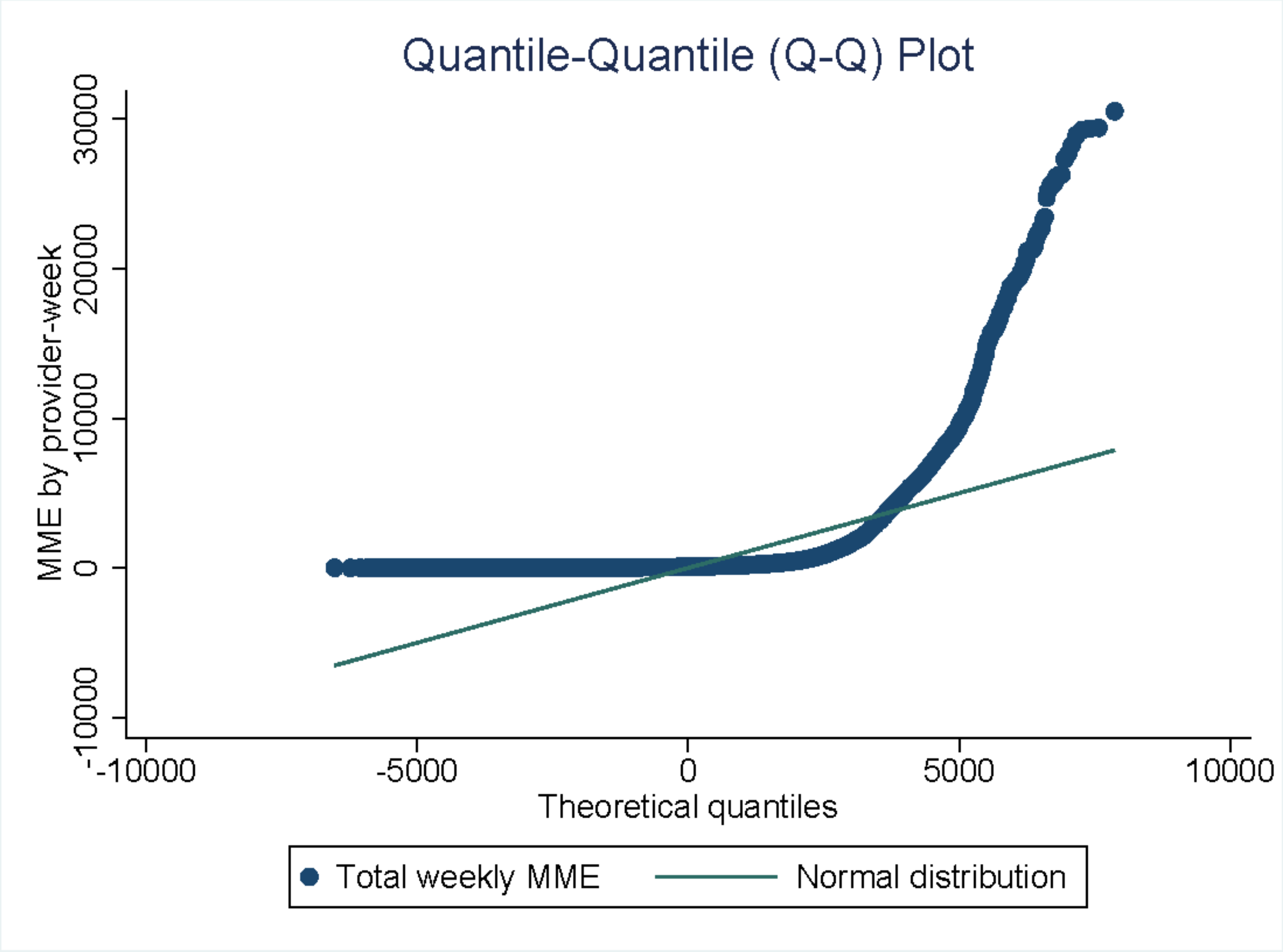

A quantile-quantile (q-q) plot showing weekly morphine milligram equivalent (MME) fails the normality assumption.

Fig S2

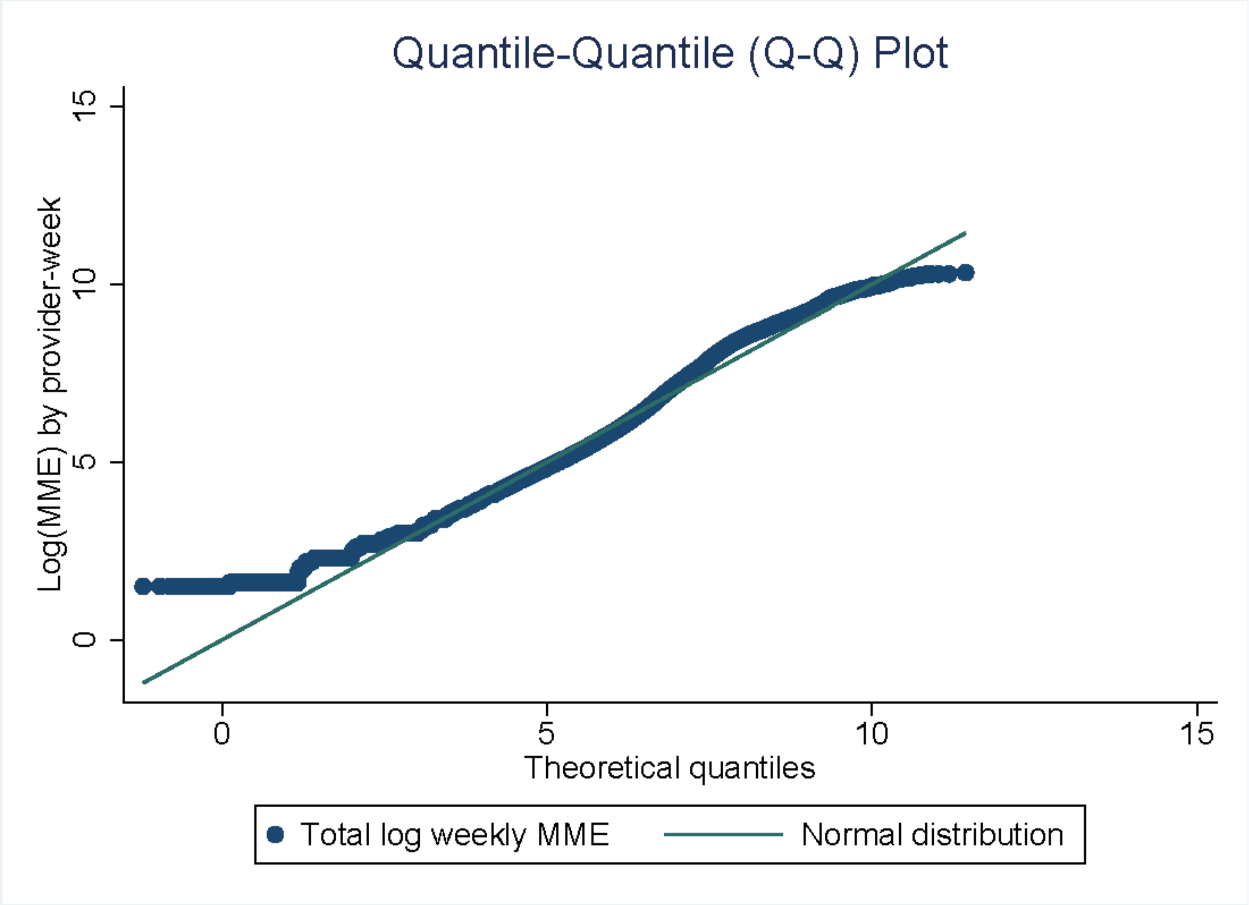

A q-q plot showing log weekly MME is similar to a normal distribution.

Table S1a. Model 1 log(MME) coefficients. SE, standard error.

Parameter	Coefficient[1]	SE	t	Two-sided P value	[95% confidence interval]	
Letter	-0.15	0.3	-0.52	0.601	-0.74	0.43
Post-intervention	-0.42	0.02	-18.16	<0.001	-0.47	-0.38
Letter*post-intervention	-0.14	0.03	-4.03	<0.001	-0.2	-0.07
Constant	3.47	0.2	16.94	<0.001	3.07	3.87
Decedent[2]	0.97	0.48	-	-	0.37	2.56
Decedent(Prescriber)[2]	7.76	0.61	-	-	6.64	9.06

[1]Censored, mixed linear regression testing two-sided hypothesis that change in pre-to-post MME does not differ by study arm.
[2]Variances for random effects.

Table S2a. Pre-intervention, per-clinician total MME outliers by study arm and decedent group.

Characteristic	No outlier	**Outlier[1]**	Chi-square[2]	Two-sided P value
Letter				
Comparator	219 (90.87%)	22 (9.13%)		
Standard	252 (93.68%)	17 (6.32%)	1.42	0.233
Decedent group				
One decedent	432 (92.11%)	37 (7.89%)		
Multiple decedents	39 (95.12%)	2 (4.88%)	0.48	0.759

[1]Tukey's fences: Q3 + 1.5*(IQR) or Q1 – 1.5*(IQR).
[2]Chi-square, or in case of cell counts less than ten, Fisher's Exact testing two-sided hypothesis that proportion of clinicians with outlier total MME does not differ by study arm or number of decedents.

Table S3a. Model 1 three-way interaction log(MME) coefficients. SE, standard error.

Parameter	Coefficient[1]	SE	t	P value	[95% confidence interval]	
Letter	-0.07	0.31	-0.23	0.82	-0.67	0.53
Post-intervention	-0.43	0.02	-17.48	0	-0.48	-0.38
Letter*post-intervention	-0.11	0.04	-3.17	0.002	-0.18	-0.04
>1 Decedent	0.94	0.61	1.53	0.125	-0.26	2.15
>1 Decedent*letter	-0.91	0.99	-0.93	0.354	-2.85	1.02
>1 Decedent*post-intervention	0.05	0.08	0.61	0.541	-0.11	0.21
>1 Decedent*post-intervention*letter	-0.38	0.13	-2.88	0.004	-0.63	-0.12
Constant	3.39	0.21	16.03	0	2.97	3.8
Decedent[2]	0.94	0.48	-	-	0.34	2.58
Decedent(Prescriber)[2]	7.74	0.62	-	-	6.62	9.05

[1]Censored, mixed linear regression testing two-sided hypothesis that change in pre-to-post MME does not differ by study arm and number of decedents.
[2]Variances for random effects.

Table S4a. Included opioids and conversion factors.

Opioid	Conversion factor
Butorphanol	7
Codeine	0.15
Dihydrocodeine	0.25
Fentanyl nasal spray	0.16
Fentanyl patch	7.2
Fentanyl buccal or SL tablets, or lozenge/troche	0.13
Hydrocodone	1
Hydromorphone	4
Levorphanol tartrate	11
Meperidine hydrochloride	0.1
Methadone	3
Morphine	1
Oxycodone	1.5
Oxymorphone	3
Pentazocine	0.37
Tapentadol	0.4
Tramadol	0.1

Fig S3

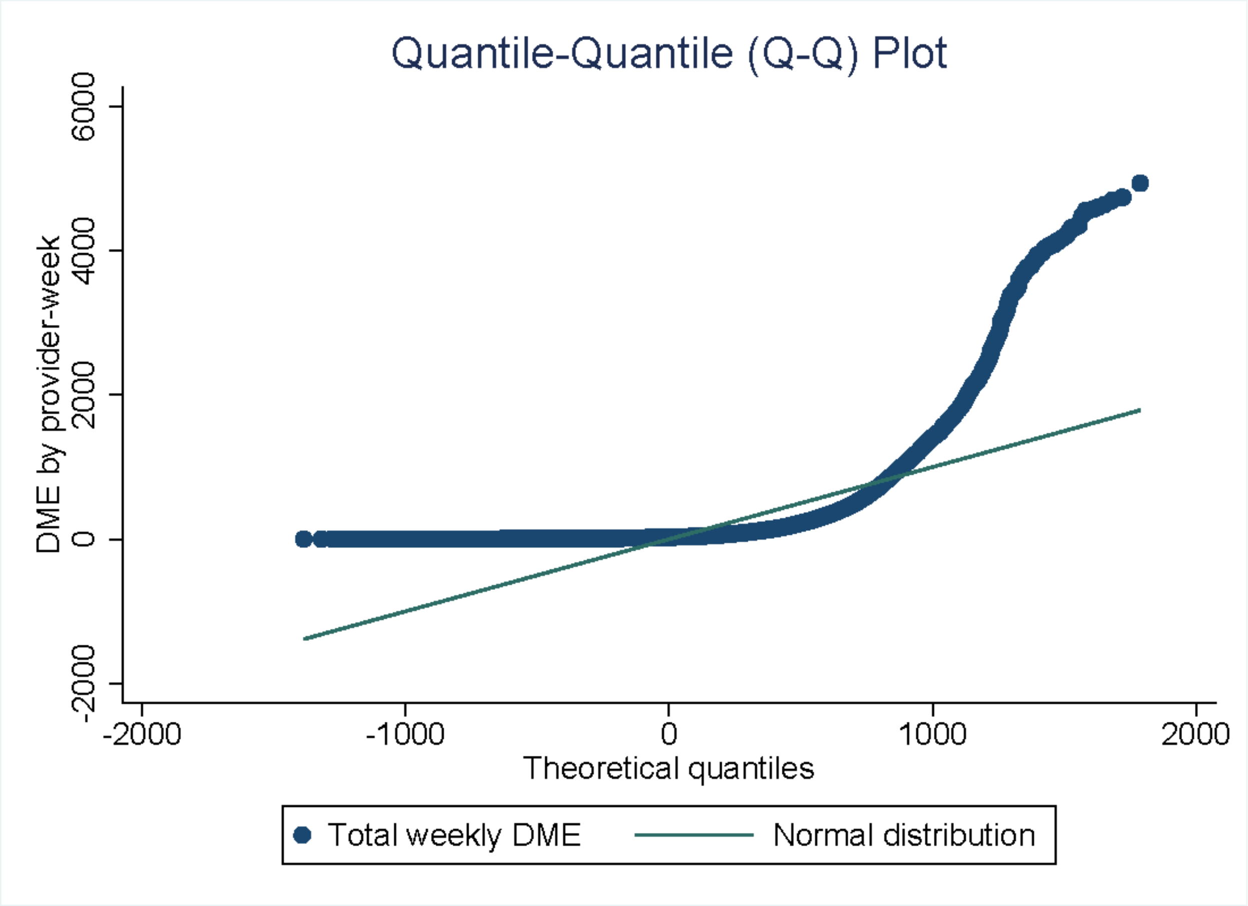

A quantile-quantile (q-q) plot showing weekly diazepam milligram equivalent (DME) fails the normality assumption.

Fig S4

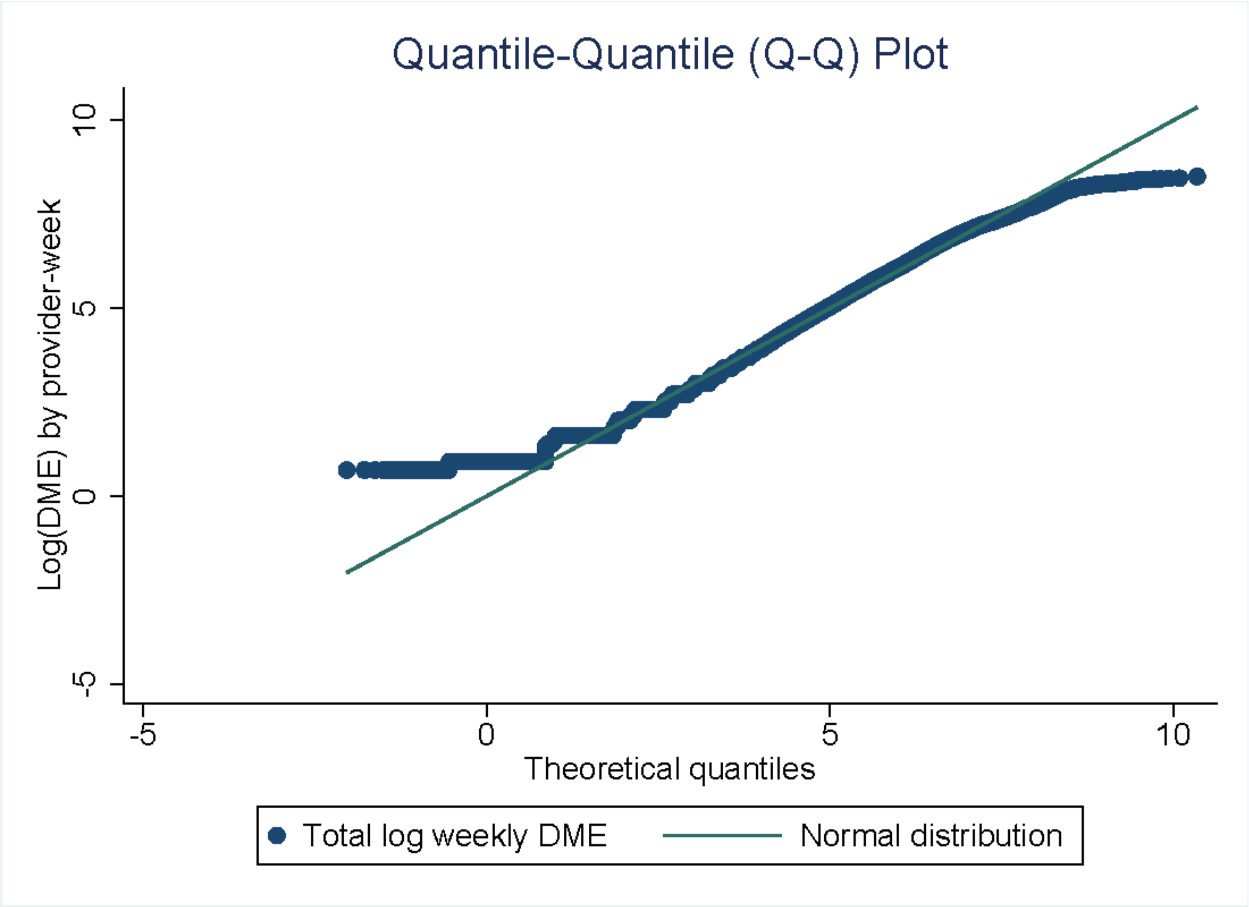

A q-q plot showing log weekly DME is similar to a normal distribution.

Table S1b. Model 1 log(DME) coefficients. SE, standard error.

Parameter	Coefficient[1]	SE	t	P value	[95% confidence interval]	
Letter	-0.23	0.29	-0.8	0.425	-0.79	0.33
Post-intervention	-0.22	0.02	-9.92	<0.001	-0.26	-0.18
Letter*post-intervention	-0.09	0.03	-2.69	<0.01	-0.15	-0.02
Constant	1.99	0.21	9.7	<0.001	1.59	2.4
Decedent[2]	0.97	0.45	-	-	0.39	2.43
Decedent(Prescriber)[2]	7.45	0.57	-	-	6.4	8.66

[1]Censored, mixed linear regression testing two-sided hypothesis that change in pre-to-post DME does not differ by study arms.
[2]Variances for random effects.

Table S2b. Pre-intervention, per-clinician total DME outliers by study arm and decedent group.

Characteristic	No outlier	Outlier[1]	Chi-square[2]	P value
Letter				
Comparator	251 (100%)	0		
Standard	273 (98.56%)	4 (1.44%)	3.65	0.125
Decedent group				
One decedent	471 (96.71%)	16 (3.21%)		
Multiple decedents	40 (97.56%)	1 (2.44%)	0.09	1.0

[1]Tukey's fences: Q3 + 1.5*(IQR) or Q1 – 1.5*(IQR).
[2]Fisher's Exact testing two-sided hypothesis that proportion of clinicians with outlier total DME does not differ by study arm or number of decedents.

Table S3b. Model 1 three-way interaction log(DME) coefficients. SE, standard error.

Parameter	Coefficient[1]	SE	t	P value	[95% confidence interval]	
Letter	-0.172	0.29	-0.6	0.552	-0.74	0.4
Post-intervention	-0.23	0.02	-9.99	0	-0.28	-0.19
Letter*post-intervention	-0.02	0.03	-0.61	0.544	-0.09	0.05
>1 Decedent	2.5	0.59	4.25	0	1.35	3.66
>1 Decedent*letter	0.13	0.95	-0.05	0.964	-1.9	1.81
>1 Decedent*post-intervention	-0.04	0.07	1.85	0.064	-0.01	0.27
>1 Decedent*post-intervention*letter	-0.82	0.11	-7.21	0	-1.05	-0.6
Constant	1.76	0.21	8.53	0	1.36	2.16
Decedent[2]	0.89	0.42	-	-	0.35	2.25
Decedent(Prescriber)[2]	7.13	0.55	-	-	6.14	8.29

[1]Censored, mixed linear regression testing two-sided hypothesis that change in pre-to-post DME does not differ by study arm and number of decedents.
[2]Variances for random effects.

Table S4b. Included benzodiazepines and conversion factors.

Benzodiazepine	Conversion factor
Alprazolam	10
Lorazepam	5
Clonazepam	10
Temazepam	0.5
Diazepam	1
Chlordiazepoxide	0.4
Triazolam	20
Oxazepam	0.33
Flurazepam	0.33
Clorazepate	0.67
Estazolam	7.5
